# Reverse vaccinology and immunoinformatics approaches for multi-epitope vaccine design against *Klebsiella pneumoniae* reveal a novel vaccine target protein

**DOI:** 10.1016/j.jgeb.2025.100510

**Published:** 2025-05-23

**Authors:** Mayada M. Elfadil, Samah Omer A. Samhoon, Moaaz M. Saadaldin, Sabah A.E. Ibrahim, Ahmed Abdelghyoum M. Mohamed, Omnia H. Suliman, Osama Mohamed, Nadzirah Damiri, Mohd Firdaus-Raih, Sofia B. Mohamed, Qurashi. M. Ali

**Affiliations:** a Bioinformatics and Biostatistics Department, National University Biomedical Research Institute, National University-Sudan, Khartoum, Sudan; b Al Neelain University Faculty of Medicine Biochemistry and Molecular Biology Department, Sudan; c Al-Neelain Institute for Medical Research, Sudan; d Department of Medicine & Surgery, Dubai Medical University, Dubai, United Arab Emirates; e Department of Molecular Biology, National University Biomedical Research Institute, National University-Sudan, Khartoum, Sudan; f Department of Applied Physics, Faculty of Science and Technology, Universiti Kebangsaan Malaysia, 43600 UKM Bangi, Selangor, Malaysia; g National University-Sudan, Khartoum, Sudan

**Keywords:** Immunoinformatics, Reverse Vaccinology, *K. pneumoniae*, Multi-Epitope Vaccine, Iron Acquisition Proteins, FyuA, IucA/IucC, TLR4 binding, Computational vaccine design, Antibiotic resistance

## Abstract

*Klebsiella pneumoniae* (*K. pneumoniae*), a Gram-negative pathogen, is a leading cause of hospital-acquired infections in Sudan and worldwide. The emergence of multidrug-resistant (MDR) strains has severely limited treatment options, underscoring the urgent need for an effective vaccine. In this study, we employed reverse vaccinology and immunoinformatics to design a novel multi-epitope vaccine targeting the hypervirulent NUBRI-K strain. Two conserved, non-host homologous iron acquisition proteins, IucA/IucC and FyuA, were prioritized as targets. The vaccine construct integrates six B-cell, six cytotoxic T lymphocyte (CTL), and six helper T lymphocyte (HTL) epitopes, linked by optimized spacers and fused to a β-defensin adjuvant. Computational analyses confirmed strong antigenicity (1.0429), non-allergenicity, and favorable solubility (0.477). Molecular docking revealed high-affinity binding to Toll-like receptor 4 (TLR4) (−278.22 kcal/mol), stabilized by eight hydrogen bonds and two salt bridges. Structural validation showed that 91 % of residues were located in favored regions of the Ramachandran plot. Additionally, CABSflex 2.0 dynamics analysis confirmed stable vaccine–TLR4 interactions, with minimal residue-level fluctuations (RMSF <1.5 Å), indicating conformational stability of the complex. In silico immune simulations predicted potent humoral and cellular responses, including elevated IgG/IgM titers, T-cell proliferation, and IFN-γ secretion. The construct was further optimized for mammalian expression, achieving an ideal GC content (48.27 %) and a codon adaptation index (CAI) of 1.0, facilitating efficient in silico cloning into the pcDNA3 vector. By targeting conserved iron acquisition systems, this vaccine candidate presents a promising strategy to combat antibiotic-resistant K. pneumoniae while minimizing selective pressure. Future in vitro and in vivo studies are warranted to validate its immunogenicity and protective efficacy*.*

## Graphical abstract

**Figure:**
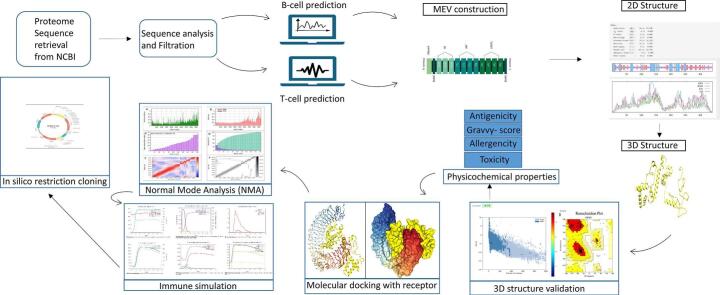

## 1 Introduction

*K. pneumoniae*, a Gram-negative pathogen, is a leading cause of invasive infections worldwide, including pneumonia, bloodstream infections, and urinary tract infections. Its capacity to cause severe disease, combined with escalating antibiotic resistance, has rendered *K. pneumoniae* a significant public health threat. It is recognized as one of the six most critical multidrug-resistant (MDR) bacterial pathogens alongside *Staphylococcus aureus*, *Pseudomonas aeruginosa*, and *Acinetobacter baumannii*. *K. pneumoniae* has evolved from being primarily a hospital-acquired pathogen to an increasing burden in community settings.^1^ The rapid spread of MDR strains, particularly those producing extended-spectrum β-lactamases (ESBLs) and *K. pneumoniae* carbapenemases (KPCs), has severely limited treatment options, leaving clinicians with few effective therapies. This challenge is particularly acute in neonates, where *K. pneumoniae* infections are associated with high fatality rates, especially in low- and middle-income countries (LMICs).^2^ The BARNARDS network reports that *K. pneumoniae* accounts for 10 % of all neonatal sepsis-related deaths in LMICs, underscoring its devastating impact on vulnerable populations.^3^ Globally, MDR *K. pneumoniae* is responsible for approximately 50 % of severe healthcare-associated infections, with resistance to last-line antibiotics such as carbapenems further complicating treatment.^4^ In Sudan, the healthcare system faces significant challenges due to the high prevalence of carbapenem-resistant *K. pneumoniae* strains. These infections contribute to prolonged hospital stays, increased healthcare costs, and elevated mortality rates. Alarmingly, recent studies indicate that a substantial proportion of clinical *K. pneumoniae* isolates in Sudan produce ESBL enzymes, further narrowing the available therapeutic arsenal.^4^

Genomic analysis of the hypervirulent NUBRI-K strain has revealed 131 virulence genes, including 34 associated with iron acquisition-a critical factor for bacterial survival and pathogenicity-as well as 24 antibiotic resistance genes conferring resistance to aminoglycosides, β-lactams, fluoroquinolones, and fosfomycin.^5^ The increasing prevalence of antibiotic resistance highlights the urgent need for alternative strategies, such as preventive vaccines, to combat *K. pneumoniae* infections. Traditional vaccine development, although successful against some pathogens, is often time-consuming, costly, and less effective against rapidly evolving bacteria like *K. pneumoniae*. Reverse vaccinology, a genome-based approach, presents a promising alternative by enabling the rapid identification of vaccine candidates through computational analysis of the pathogen’s proteome. This method allows the prediction of antigens capable of eliciting strong immune responses, while immunoinformatics tools refine vaccine design by mapping B-cell and T-cell epitopes to ensure high immunogenicity and broad population coverage.^6^ Despite *K. pneumoniae* being designated a critical priority pathogen by the World Health Organization (WHO), no licensed vaccine is currently available to prevent or treat its infections.^7^

Previous vaccine efforts have primarily focused on outer membrane proteins (e.g., OmpA, OmpK36), capsular polysaccharides (CPS), and lipopolysaccharides (LPS). While these antigens have shown promise in preclinical models, their high genetic variability and potential for immune evasion limit their ability to provide broad and durable protection.^8^ In contrast, targeting conserved virulence factors, such as iron acquisition systems, offers a strategic advantage. Iron is essential for bacterial survival and virulence, and *K. pneumoniae* relies on siderophore-associated proteins such as FyuA and IucA/IucC to scavenge iron from the host. These proteins are highly conserved across diverse *K. pneumoniae* strains, reducing the risk of immune escape and making them attractive vaccine targets.^9^ Notably, FyuA has been validated as a vaccine antigen in Escherichia coli models, demonstrating its immunogenic potential and providing strong rationale for its inclusion in a *K. pneumoniae* vaccine.^10^ Moreover, targeting iron acquisition systems helps reduce selective pressure on antibiotic resistance genes, offering a key advantage over traditional vaccine strategies.^10^ This study leverages reverse vaccinology and immunoinformatics to design a novel multi-epitope vaccine targeting the hypervirulent NUBRI-K strain of *K. pneumoniae*. By focusing on conserved iron acquisition proteins, the proposed vaccine aims to elicit robust and long-lasting immunity while minimizing the potential for resistance development. The vaccine construct incorporates B-cell, cytotoxic T lymphocyte (CTL), and helper T lymphocyte (HTL) epitopes, linked by optimized spacers and fused to a β-defensin adjuvant to enhance immune stimulation. Computational analyses confirm the vaccine’s high antigenicity, non-allergenicity, and solubility, while molecular docking studies demonstrate strong binding affinity to Toll-like receptor 4 (TLR4), a key mediator of innate immunity. Immune simulations predict robust humoral and cellular responses, suggesting the vaccine’s potential to elicit effective protection against *K. pneumoniae* infections.

The proposed vaccine is particularly relevant for low-resource settings such as Sudan, where the burden of MDR *K. pneumoniae* infections is high and access to effective treatments remains limited. By addressing the urgent need for a preventive vaccine, this study contributes to global efforts to combat antimicrobial resistance and reduce the morbidity and mortality associated with *K. pneumoniae*. Furthermore, it highlights the promise of computational vaccine design in addressing emerging infectious threats, offering new insights into *K. pneumoniae* immunogenicity and paving the way for future experimental validation and clinical development.

## 2 Materials and methods

**Fig. 1:**
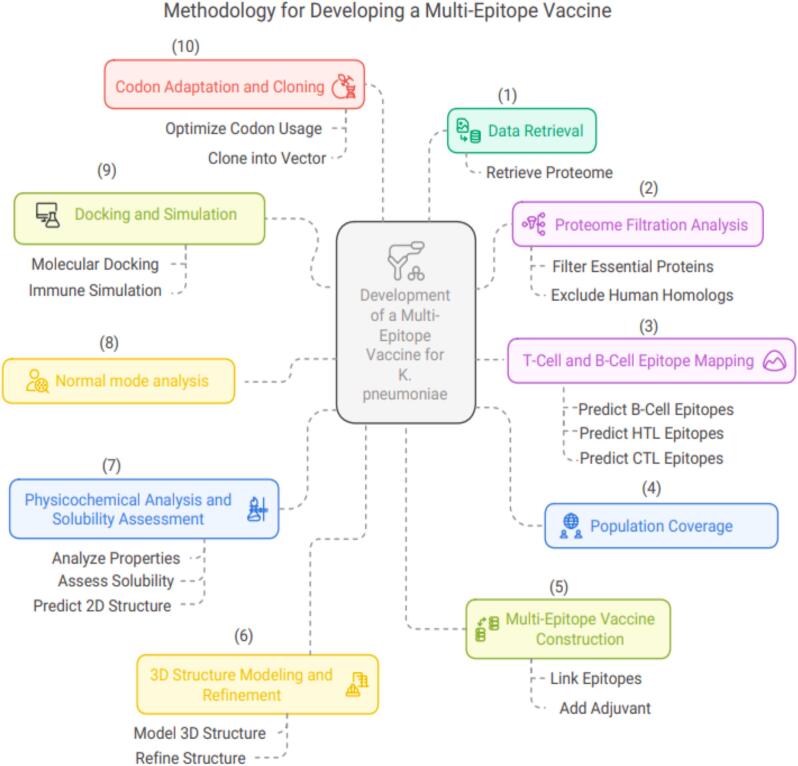
A stepwise computational workflow for developing a multi-epitope vaccine against Klebsiella pneumoniae. The process includes proteome retrieval, essential protein filtering, epitope prediction, population coverage analysis, vaccine construction with adjuvant, structural modeling, physicochemical and flexibility assessment, docking with immune receptors, immune simulation, and final codon optimization with in silico cloning into the pcDNA3 vector.

The study was divided into two phases. In the first phase, the *K. pneumoniae* proteome was subjected to a series of filtration processes utilizing reverse vaccinology and subtractive genomic methods to identify potential protein candidates for vaccine design. The second phase involved the construction, evaluation, and assessment of the multi-epitope vaccine using predicted B and T cell epitopes (Fig. 1).

### 2.1 Data retrieval

The 5,607 proteins of *K. pneumoniae* strain NUBRI-K (ST14) were retrieved from NCBI using the GenBank assembly accession number GCA_004790705.1. This strain was selected based on its high virulence potential and extensive repertoire of resistance genes, as identified in our previous whole-genome sequencing project conducted at the National University Biomedical Research Institute (NUBRI). Notably, it represents the first publicly available genome of a multidrug-resistant *K. pneumoniae* strain isolated in Sudan, making it a uniquely relevant model for studying regional epidemiology and guiding vaccine development in low-resource settings.

The NUBRI-K genomic profile includes multiple virulence determinants particularly iron acquisition systems and numerous antimicrobial resistance genes, underscoring the pathogen's clinical significance and its contribution to the growing burden of untreatable infections. These attributes make it a representative and clinically meaningful strain for reverse vaccinology-based vaccine design.^5^

### 2.2 Proteome filtration analysis

A default essentiality threshold of 0.24 was selected in Geptop 2.0 to balance sensitivity and specificity. This value ensures the inclusion of key survival proteins while minimizing false positives. A stricter threshold could inadvertently exclude essential virulence factors, whereas a more lenient one might introduce non-essential proteins, potentially compromising vaccine efficacy.^11^

Subcellular localization was determined using PSORTb v3.0.2, which classified the filtered proteins based on their predicted cellular compartments.^12^ Virulent proteins were then identified using the VirulentPred server, focusing specifically on essential exoproteome and secretome components.^13^

To avoid autoimmune responses, the identified virulence proteins were aligned against the human proteome using blastp, and those with more than 35 % sequence identity to human proteins were excluded.^14^ Transmembrane helices were predicted using the TMHMM server,^15^ and molecular weights were estimated using ProtParam.^16^ Only proteins with molecular weights < 110 kDa and < 1 transmembrane helix were selected for antigenicity evaluation using VaxiJen 2.0 (http://www.ddg-pharmfac.net/vaxijen/VaxiJen.html). A VaxiJen score threshold of 0.4 was applied to identify antigenic proteins, balancing the inclusion of potentially immunogenic candidates with the exclusion of weakly antigenic ones.^17^

All selected proteins were further screened for toxicity using ToxinPred, and only non-toxic candidates were retained. Proteins meeting the criteria of being non-toxic, non-allergenic, and possessing strong antigenicity were subjected to epitope prediction.

To ensure broad-spectrum efficacy and relevance, a conservation analysis was performed on 100 *K. pneumoniae* IucA/IucC and FyuA protein sequences. These sequences were carefully selected from the NCBI protein database to represent a diverse range of geographic origins, clinical sources, and sequence types (STs), including multidrug-resistant and hypervirulent strains. Reference sequences (WP_236946543.1 for IucA/IucC and WP_225322595.1 for FyuA) from the NCBI RefSeq database were included in the multiple sequence alignment, which was conducted using Clustal W in the BioEdit software. Proteins showing less than 90 % sequence conservation were excluded. Each candidate was further evaluated against representative strains from different K. pneumoniae pathotypes to confirm broad-spectrum applicability. This rigorous selection process ensured that only highly conserved, antigenic, non-toxic, and non-allergenic proteins were included in the final vaccine construct, thereby maximizing safety and immunogenic potential.^18^

### 2.3 B-Cell, T-Cell, and HTL epitope prediction and evaluation

#### 2.3.1 B-cell epitopes

B-cell epitopes were predicted based on their potential to interact with B-cell lymphocyte (BCL) receptors, which are essential for initiating strong humoral immune responses. The ABCpred server was utilized with a threshold of 0.5, corresponding to an accuracy of 65.93 %, to balance sensitivity and specificity. A window length of 16 amino acids was used to identify epitopes with a high likelihood of activating B-cell responses.^14^

#### 2.3.2 Cytotoxic T-cell (CTL) epitopes

CD8 + T-cell (cytotoxic T lymphocyte, CTL) epitopes were identified using the IEDB MHC Class I binding prediction tool (https://www.iedb.org).^19^ Predictions were made using the Artificial Neural Network (ANN) algorithm, focusing on 9-mer peptides that demonstrated high-affinity binding to 27 commonly occurring HLA alleles. Peptides with IC50 values ≤ 100 nM were prioritized, as these are associated with strong binding to MHC-I molecules and are more likely to elicit potent cytotoxic immune responses.^20^

#### 2.3.3 Helper T-cell (HTL) epitopes

HTL (CD4 + ) epitopes were predicted using the IEDB MHC Class II binding tool, covering HLA-DP, HLA-DQ, and HLA-DR loci. The NN-align method was employed to identify peptides based on their percentile ranks and IC50 values. Epitopes with IC50 ≤ 50 nM were selected, reflecting their strong binding potential to MHC-II molecules and their ability to effectively activate helper T-cell responses.^21^

#### 2.3.4 Antigenicity, allergenicity, and toxicity assessment

All predicted epitopes were evaluated for antigenicity using VaxiJen 2.0, applying a threshold of 0.4 to confirm immunogenic potential. Allergenicity was assessed using AllerTOP v2.0, and toxicity was screened using ToxinPred, a machine-learning-based classifier designed to identify and eliminate toxic peptides.^22^

#### 2.3.5 Cytokine induction analysis

To evaluate the immunomodulatory capabilities of HTL epitopes, their potential to induce cytokine responses was analyzed. The IFNepitope server was used to predict gamma interferon (IFN-γ) induction, a critical cytokine in Th1-mediated immunity. Additionally, IL4pred and IL10pred servers were employed to assess the capacity of selected epitopes to induce interleukin-4 (IL-4) and interleukin-10 (IL-10), which are essential for regulating immune homeostasis and anti-inflammatory responses.

This integrative and rigorous selection strategy ensured the identification of epitopes with high immunogenic potential, broad HLA population coverage, and minimal risk of adverse effects, thereby optimizing the safety and efficacy of the proposed multi-epitope vaccine candidate.^23^

### 2.4 Epitope conservation analysis, population coverage, and autoimmunity screening

The predicted CD8+ (CTL), CD4+ (HTL), and B-cell (BCE) epitopes were rigorously evaluated for sequence conservation. Only epitopes that were fully conserved across multiple K. pneumoniae strains-based on multiple sequence alignment-were retained in the final vaccine construct. This strategy ensured the inclusion of epitopes derived from highly conserved antigenic regions, thereby minimizing the risk of strain-specific immune evasion and enhancing cross-strain protective efficacy.

Considering the variability in HLA allele distribution across different ethnic and geographic populations, the IEDB Population Coverage Tool^24^ was employed to assess the global and regional prevalence of the selected CTL and HTL epitopes based on HLA genotype frequencies.^25^ The analysis was conducted independently for MHC class I and MHC class II epitopes, followed by a combined population coverage assessment to evaluate the cumulative immune response potential across diverse human populations.

To minimize the risk of autoimmune responses, all selected epitopes were aligned against the human proteome using blastp. Epitopes exhibiting ≥35 % sequence identity to human proteins were flagged as potentially cross-reactive and excluded from further consideration. This stringent exclusion criterion was adopted to ensure that only non-homologous epitopes were incorporated into the final vaccine construct, thereby reducing the risk of self-reactivity and autoimmune complications.^26^

### 2.5 Multi-epitope vaccine construction

Epitope mapping was conducted to identify optimal candidates for Cytotoxic T Lymphocyte (CTL), Helper T Lymphocyte (HTL), and B-cell Lymphocyte (BCL) epitopes to be incorporated into the multi-epitope vaccine construct. To ensure proper immunological processing and presentation, the selected epitopes were joined using specific linkers optimized for immune recognition:

• KK (Lys-Lys) linker was used to connect B-cell epitopes, enhancing their flexibility and ensuring optimal antigen exposure to B-cell receptors.
• AAY (Ala-Ala-Tyr) linker was used to enhance the immunogenicity of the epitope vaccine.
• GPGPG (Gly-Pro-Gly-Pro-Gly) linker was used between HTL epitopes to promote MHC class II binding and enhance helper T-cell responses. This linker helps in the effective presentation of the epitopes to the immune system, improving the overall immunogenicity of the vaccine.
• EAAAK (Glu-Ala-Ala-Ala-Lys) linker was introduced between the adjuvant and the multi-epitope construct to form a rigid α-helix, maintaining structural stability and minimizing steric interference between functional domains.^27^

To enhance the immunogenic potential of the vaccine, β-defensin (UniProt ID: P81534) was incorporated as an adjuvant at the N-terminus. This antimicrobial peptide was selected for its well-documented role in:

• Activating dendritic cells and promoting efficient antigen presentation,
• Stimulating innate immune responses, and
• Inducing pro-inflammatory cytokines and chemokines to recruit and activate immune cells.

The β-defensin adjuvant was fused to the vaccine construct via the EAAAK linker, ensuring spatial and functional segregation from the antigenic epitopes.

Furthermore, a C-terminal 6 × His tag was appended to facilitate downstream purification and identification of the recombinant protein using affinity chromatography and immunodetection techniques.^28^ In the current design, the multi-epitope vaccine construct does not include a signal peptide or secretion tag.

### 2.6 Analysis of physicochemical characteristics, assessment of protein solubility, and estimation of secondary structure

The physicochemical properties of the designed multi-epitope vaccine were comprehensively analyzed using the ProtParam tool available on the ExPASy server. This analysis included evaluation of molecular weight, theoretical isoelectric point (pI), instability index, aliphatic index, and GRAVY (Grand Average of Hydropathicity) score. These parameters are crucial for assessing protein stability, solubility, and expression potential.^20^ The GRAVY score provides insight into the hydrophobic or hydrophilic nature of the vaccine construct. A lower GRAVY value indicates increased hydrophilicity, which is favorable for solubility and epitope accessibility, thereby enhancing immune recognition. Conversely, highly hydrophobic proteins are more prone to aggregation, which may negatively impact stability and purification efficiency.

To evaluate the antigenic potential, the construct was analyzed using VaxiJen v2.0, an alignment-free prediction tool that effectively distinguishes between self and non-self antigens, confirming the ability of the construct to elicit a robust immune response. In parallel, SOLpro from the SCRATCH suite was used to predict the solubility of the vaccine upon overexpression in *E. coli*, an important factor for experimental validation and recombinant protein production.^19^ The secondary structure of the vaccine protein-comprising alpha helices, beta sheets, and random coils-was predicted using the Self-Optimized Prediction Method (SOPMA). This analysis provides foundational insight into the protein’s structural organization, which directly influences stability and immunogenic presentation.^22^

Following the tertiary structure prediction and refinement, B-cell conformational epitopes (discontinuous epitopes) were mapped using ElliPro, a tool integrated in the IEDB suite. ElliPro predicts antibody-accessible regions based on protein geometry, solvent accessibility, and structural flexibility.^29^ A minimum score threshold of 0.5 and a maximum distance of 6 Å were employed as selection criteria. The rationale behind the 6 Å distance cutoff is its ability to balance specificity and sensitivity, allowing for the inclusion of flexible, structurally relevant antibody-binding sites. Furthermore, the Area Under the Curve (AUC) score of 0.732 suggests moderate to high reliability of ElliPro predictions, supporting the robustness of the predicted conformational epitopes. The use of stringent and biologically relevant cutoffs ensures methodological rigor and enhances the reproducibility of the findings.

### 2.7 Tertiary structure prediction, refinement, and validation

Tertiary structure prediction of the designed multi-epitope vaccine is a critical step in evaluating its stability, structural integrity, and antigenicity.^27^ The initial 3D structure of the vaccine construct was predicted using 3Dpro, a tool available through the SCRATCH protein predictor server.^28^ 3Dpro was selected for its de novo modeling capability, making it particularly suitable for novel proteins that lack homologous structural templates. Unlike homology-based modeling approaches, 3Dpro generates structural predictions solely from sequence data, thereby enabling accurate modeling even in the absence of evolutionary or structural analogs.

To improve the quality of the predicted model—specifically in terms of folding accuracy, structural stability, and minimization of steric clashes—the structure was refined using GalaxyRefine.^29^ This tool optimizes local structure through repeated side-chain repacking and overall structural relaxation, enhancing the physical plausibility of the model.

The refined structure underwent Ramachandran plot analysis to assess the ϕ (phi) and ψ (psi) dihedral angles of amino acid residues. The results confirmed that the majority of residues were located within favored and allowed regions, indicating a geometrically sound conformation with minimal stereochemical outliers.

Further validation of the model's structural quality was conducted using ProSA-web, which provides a Z-score reflecting the overall model quality by comparing its energy distribution with that of experimentally determined protein structures from the Protein Data Bank.^30^ The Z-score fell within the range typical of native proteins of comparable size, supporting the structural authenticity and reliability of the vaccine model.

### 2.8 Molecular docking of designed vaccine with TLR-4 and molecular docking and binding affinity analysis

To evaluate the interaction between the designed multi-epitope vaccine and its innate immune receptor, molecular docking was performed against Toll-like receptor 4 (TLR4). We chose TLR4 for docking analysis due to its well-characterized extracellular domain, availability of high-resolution structural data and a key pattern recognition receptor involved in immune responses to Gram-negative pathogens such as *Klebsiella pneumonia*.^31^ TLR4 (PDB ID: 4G8A) was selected due to its well-characterized extracellular domain and proven role in recognizing pathogen-associated molecular patterns (PAMPs). Prior to docking, the TLR4 structure was preprocessed in UCSF Chimera by removing all non-protein molecules, including water and heteroatoms, to ensure accurate interaction modeling. Protein-protein docking was performed using the ClusPro web server, which ranks docked complexes based on cluster size and energy minimization scores. The top-ranked model—identified by the largest cluster size and lowest binding energy—was selected for further analysis. To increase confidence in the docking results, cross-validation was conducted using the H-DOC server, which employs distinct energy-based scoring functions to assess protein–protein interactions. The binding affinity (ΔG, kcal/mol) and dissociation constant (Kd, M) of the vaccine–TLR4 complex were estimated using the PRODIGY web server, which integrates both empirical and computational data to predict the thermodynamic stability of protein complexes. To visualize molecular interactions, the docked complex was analyzed using UCSF ChimeraX, focusing on key binding residues, interface topology, and interaction networks. To refine the complex further and improve docking accuracy, HADDOCK 2.4 was utilized. This high-ambiguity-driven docking platform refines molecular interfaces through flexible side-chain adjustments and energy optimization algorithms. Additionally, PDBsum was employed to provide a detailed analysis of the vaccine–TLR4 complex, including hydrogen bond interactions, salt bridges, and interface residue mapping, offering a comprehensive understanding of the molecular determinants underlying the binding interaction.^32^

### 2.9 Molecular simulation

To investigate the stability, flexibility, and dynamic behavior of the vaccine–TLR4 complex, Normal Mode Analysis (NMA) was conducted. This approach enables the exploration of large-scale conformational motions that occur near the equilibrium state, which are critical for maintaining structural integrity and functional immune recognition. Due to its computational efficiency, NMA is especially advantageous for assessing macromolecular flexibility in protein–protein interactions.

The PDB-formatted docked complex was analyzed using the iMODS server (https://imods.chaconlab.org/). iMODS employs internal coordinate-based NMA, utilizing dihedral angles rather than Cartesian coordinates, which enhances the precision of predicted collective motions and deformation patterns within protein complexes.

The iMODS analysis provided a comprehensive set of graphical and quantitative outputs, including:

Deformability Plot: Highlights localized flexible regions within the complex, suggesting potential movement zones critical for interaction adaptability.

B-factor Plot: Simulates atomic displacement analogous to experimental temperature factors, providing insights into residue-level thermal motion.

Eigenvalue: Reflects the energy required for structural deformation; a lower eigenvalue indicates greater flexibility and a lower energy cost for conformational changes.

Variance and Covariance Mapping: Illustrates correlated and anti-correlated motions between residue pairs, shedding light on cooperative dynamics and potential allosteric effects.

Elastic Network Model: Displays inter-residue connections via spring-like interactions, evaluating the mechanical stability and rigidity of the vaccine–receptor complex.

Together, these NMA parameters offer vital insights into the structural resilience and biological plausibility of the vaccine–TLR4 complex, reinforcing its suitability for stimulating robust innate immune responses.^29^

### 2.10 Immune simulation of the designed vaccine

To evaluate the immunogenic potential and host immune response to the designed multi-epitope vaccine, in silico immune simulations were performed using the C-ImmSim server (https://kraken.iac.rm.cnr.it/C-IMMSIM/). C-ImmSim integrates a position-specific scoring matrix (PSSM) with machine learning algorithms to simulate complex immune interactions between antigenic epitopes and host immune components in a mammalian model.

The simulation environment mimics the function of three primary immune system compartments:

Bone Marrow: Site of hematopoiesis where progenitor stem cells differentiate into various lymphoid and myeloid cells, initiating the immune response.

Thymus: Central to T-cell development and negative selection, ensuring immune self-tolerance and elimination of autoreactive clones.

Tertiary Lymphoid Organs (e.g., Lymph Nodes): Key sites for antigen processing, presentation, and adaptive immune activation, including T and B cell clonal expansion.

A prime-boost strategy was implemented by scheduling three vaccine doses at time steps 1, 84, and 168 (corresponding to weeks 0, 4, and 8), effectively simulating a longitudinal vaccination protocol. Each time step in the simulation equates to 8 h of real time. Importantly, lipopolysaccharide (LPS) was excluded from the immunization to avoid nonspecific immune activation and to focus on the vaccine-specific response.

The simulation outputs included dynamic profiling of key immunological parameters, such as:

Primary and secondary immune responses (IgM, IgG1, IgG2, T-cell populations, cytokine profiles).

Memory cell formation and clonal expansion

Cytokine and interleukin levels, indicating immune activation intensity.

Additionally, immune response diversity was quantified using the Simpson Index (D), a statistical measure of repertoire heterogeneity. A higher Simpson Index value indicates greater clonal diversity, suggesting a more robust and adaptable immune response.^33^

### 2.11 Coarse-Grained dynamics simulation of the target–vaccine complex

To assess the structural stability and flexibility of the vaccine–TLR4 complex, coarse-grained molecular dynamics (MD) simulations were initially conducted using the CABS-flex 2.0 server (https://biocomp.chem.uw.edu.pl/CABSflex2, accessed on 4 October 2022). CABS-flex offers an efficient platform for modeling protein conformational flexibility and detecting local residue fluctuations with high accuracy while significantly reducing computational cost.

Root Mean Square Fluctuation (RMSF) profiles were computed under default restraint parameters, enabling identification of flexible regions and conformational hotspots within the complex. These fluctuations provided residue-level insight into dynamic behavior, allowing the evaluation of structural elements most critical for maintaining interaction stability and antigenic exposure.

To complement the coarse-grained approach, an all-atom force field-based MD simulation was performed to capture atomic-level dynamics of the docked complex in an explicit aqueous environment. The simulation was run for 10 ns, using standard parameters:

Minimum and maximum distance restraints of 3.8 Å and 8.0 Å, respectively.

A restraint gap of 3, representing the minimum sequence separation between restrained residue pairs.

The RMSF data from the all-atom simulation were plotted and compared to coarse-grained results to validate the stability and rigidity of the interface. Residues exhibiting low atomic fluctuations were considered structurally stable and potentially important for maintaining vaccine–receptor binding fidelity.

Together, these simulations provided a robust dynamic assessment of the vaccine–TLR4 interaction, highlighting conformational consistency, interface rigidity, and regions of structural plasticity essential for downstream vaccine efficacy.^34^

### 2.12 Codon adaptation, translation and cloning

To facilitate efficient expression of the multi-epitope vaccine in mammalian host systems, the amino acid sequence was reverse-translated and codon-optimized using the JCat tool (https://www.jcat.de). Codon optimization is a critical step in synthetic gene design, aiming to maximize translational efficiency, mRNA stability, and protein yield by aligning the codon usage bias with that of the host organism.

The optimization process yielded a GC content of 48.27 %, which falls within the optimal range of 30–70 % for mammalian cells, ensuring efficient transcription, stable mRNA structure, and reduced secondary structure formation. A balanced GC content also enhances ribosome binding and minimizes the risk of transcriptional pausing. Additionally, the Codon Adaptation Index (CAI) was calculated to be 1.0, indicating perfect adaptation to the codon usage preference of mammalian systems, thereby maximizing translation potential and minimizing the risk of premature translational arrest.

To enable downstream cloning, restriction sites for NcoI and XhoI were introduced at the N-terminal and C-terminal ends of the optimized cDNA sequence, respectively. The final gene construct was then subjected to in silico cloning using the GenSmart Design Tool (https://www.genscript.com/gene-and-plasmid-construct-design.html). The optimized gene was successfully inserted into the pcDNA3 mammalian expression vector under the control of the cytomegalovirus (CMV) promoter, a well-characterized strong promoter ensuring high-level transcription in mammalian cells. XboI and ApaI restriction sites were used for cloning into the vector backbone, enabling seamless integration for experimental validation and transient expression studies.^35^

This optimized design ensures robust expression of the vaccine construct in mammalian systems, a key requirement for in vitro functional assays and preclinical evaluation.

## 3 Results

### 3.1 Protein vaccine candidate selection methodology

**Fig. 2:**
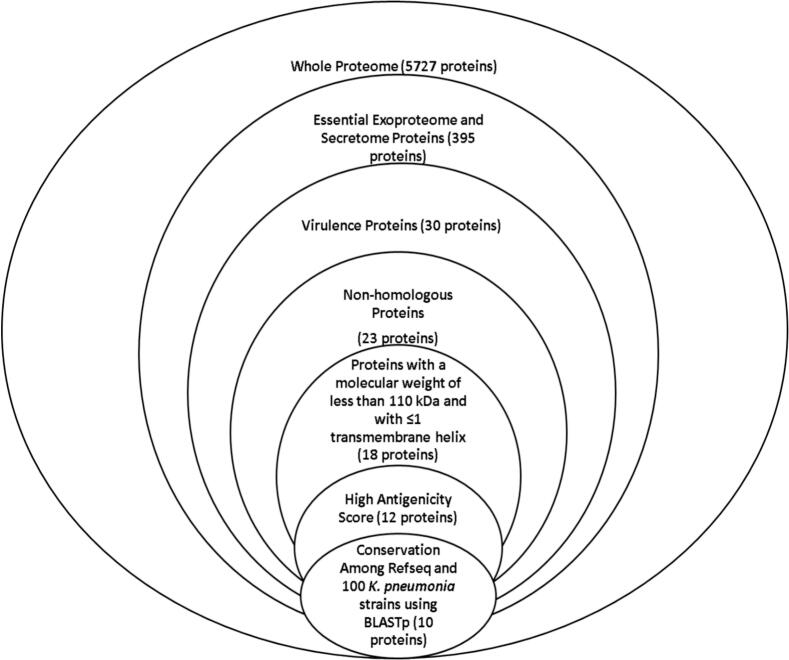
Protein filtration workflow for vaccine candidate selection. The flowchart details the sequential bioinformatic filters applied to identify optimal vaccine targets from the *K. pneumoniae* proteome. Starting with 5,607 proteins, successive filters included: essentiality scoring (<0.24), extracellular localization, virulence prediction, human non-homology (<35 % identity), transmembrane helix restriction (≤1), molecular weight cutoff (<110 kDa), and antigenicity scoring (>0.4).

The filtration pipeline used to identify potential vaccine candidates is illustrated in Fig. 2. A systematic screening strategy was implemented to prioritize proteins with high antigenicity, while ensuring that all critical selection criteria such as subcellular localization, non-homology to human proteins, and essentiality—were satisfied. This multi-step bioinformatic workflow enabled the refinement of a large proteomic dataset into a focused list of high-confidence candidates.

As a result of this analysis, the top ten proteins with the highest VaxiJen antigenicity scores were shortlisted as potential vaccine candidates (Table S1). Among these, two proteins emerged as the most promising targets:

IucA/IucC family siderophore biosynthesis protein (accession: TGP00625.1).

Siderophore yersiniabactin receptor FyuA (accession: TGP03888.1).

These two candidates demonstrated the highest antigenicity values, highlighting their potential as immunogenic components in a subunit vaccine (Table 1). To further validate their utility as broad-spectrum vaccine targets, conservation analysis was performed across 100 *K. pneumoniae* genomes. The IucA/IucC protein showed 100 % sequence identity, while FyuA displayed 99.9 % identity, confirming their conserved nature and potential efficacy across multiple *K. pneumoniae* strains (Fig. S1).

**Table 1:** The outcome of the protein filtrations conducted using various bioinformatics tools.

Protein	TMH*≤1	MW* <110 kDa	Antigenicity	Conservation
**FyuA (TGP03888.1)**	0	73.6	ANTIGEN (0.6656)	Conserved
**IucA/IucC family (TGP00625.1)**	0	65.8	ANTIGEN (0.4188)	Conserved

TMH*: Transmembrane Helix. MW*: Molecular Weight.

This robust candidate selection approach ensures that the final vaccine construct is not only antigenic but also widely applicable in combating genetically diverse strains of *K. pneumoniae*.

### 3.2 B-cell and T-cell epitope prediction

Immunoinformatic screening revealed a total of eighteen high-confidence epitopes derived from the FyuA and IucA/IucC proteins, selected based on antigenicity, conservancy, allergenicity, and toxicity profiles.

Six B-cell epitopes (each 16 amino acids in length) were identified with high antigenicity scores ranging from 0.61 to 1.76, and all were classified as non-allergenic and non-toxic (Table 2). These linear B-cell epitopes are crucial for stimulating robust humoral immune responses.

**Table 2:** Predicted B cell epitopes, their antigenicity, allergenicity and toxicity from FyuA and IucA/IucC family.

Protein	Epitope	Start position	Score	Length	Antigenicity	Allergenicity	Toxicity
FyuA	EGGVSSRDSYRSKFNL	166	0.85	16	1.7641	Non-allergen	Non-toxin
	PRYGAGSSVNGVIDTR	570	0.77	16	1.7161	Non-allergen	Non-toxin
	AQVNMGRTVGINTRID	656	0.76	16	1.2675	Non-allergen	Non-toxin
IucA/IucC	DSLPQEVRDVTARLSA	458	0.77	16	0.6163	Non-allergen	Non-toxin
	LLMQLKPVLSMSDATV	77	0.74	16	0.6972	Non-allergen	Non-toxin
	GLDNDWLPLPVHPWQW	210	0.74	16	1.2431	Non-allergen	Non-toxin

Six MHC Class I cytotoxic T lymphocyte (CTL) epitopes were predicted to have strong binding affinities to human leukocyte antigen (HLA) alleles, with IC_50_ values ranging from 3.39 to 98.38 nM. All CTL epitopes demonstrated 100 % sequence conservancy and exhibited antigenicity scores between 1.22 and 3.27 (Table 3), indicating potent cytotoxic potential across diverse strains.

**Table 3:** Predicted MHC Class I Cytotoxic T Lymphocyte (CTL) Epitopes from FyuA and IucA/IucC Proteins with Associated HLA Alleles, Antigenicity, Allergenicity, Toxicity, Conservancy, and Binding Affinity (IC50).

Protein	Epitope	HLA Alleles	antigenicity	Allergenicity	Toxicity	Conservancy (%)	Start	End	IC50
FyuA	TTDDWVFNL	HLA-A*02:06	1.7032	Non-allergen	Non-toxin	100	10	18	43.89
	YMLTDDWRV	HLA-A*02:01	1.5619	Non-allergen	Non-toxin	100	19	27	3.39
	FAPGWSWDI	HLA-A*02:06	3.2759	Non-allergen	Non-toxin	100	47	55	40.54
IucA/IucC	PQMSARFAL	HLA-B*39:01	1.2247	Non-allergen	Non-toxin	100	32	40	60.27
	VVVVPLYHL	HLA-A*02:06	1.266	Non-allergen	Non-toxin	100	53	61	98.38
	TNASRQGGL	HLA-A*68:02	1.7343	Non-allergen	Non-toxin	100	52	60	83.7

Six MHC Class II helper T lymphocyte (HTL) epitopes were also identified, each showing complete conservancy, non-toxicity, and non-allergenicity, with antigenicity scores ranging from 0.57 to 1.06. These HTL epitopes exhibited strong binding affinities (IC_50_: 5.5–21.4 nM) to multiple MHC II alleles and were predicted to stimulate IFN-γ and IL-4 responses, but not IL-10 (Table 4). This cytokine profile supports a balanced Th1/Th2 immune response, crucial for both cellular and humoral immunity.

**Table 4:** Predicted MHC Class II Helper T Lymphocyte (HTL) Epitopes from FyuA and IucA/IucC Proteins with HLA Alleles, Antigenicity, Allergenicity, Toxicity, Conservancy, Cytokine Induction, and Binding Affinity (IC50).

Protein	Epitope	HLA Alleles	Antigenicity	Allergenicity	Toxicity	Conservancy (%)	IFN-γ	IL-10 Induction	IL4-inducer	Start	End	IC50
**FyuA**	LFSTISLRG	HLA-DQA1*01:02/DQB1*05:01	0.7458	Non-allergen	Non-toxin	100	Positive	Non-inducer (−0.2)	Inducer (0.33)	14	28	21.4
	YNPAVTLYV	HLA-DRB1*01:01	0.8693	Non-allergen	Non-toxin	100	Positive	Non-inducer (−0.5)	Inducer (0.27)	29	43	10.5
	MLFSTISLR	HLA-DRB1*13:01	1.0614	Non-allergen	Non-toxin	100	Positive	Non-inducer (−0.15)	Inducer (0.27)	15	29	12.4
**IucA/IucC**	FKPQIIRVV	HLA-DRB3*03:01	0.57	Non-allergen	Non-toxin	100	Positive	Non-inducer (−0.6)	Inducer (0.29)	42	56	7.8
	VVLNPVKLT	HLA-DRB3*03:01	0.6097	Non-allergen	Non-toxin	100	Positive	Non-inducer (−1.03)	Inducer (0.34)	45	59	5.5
	YRGIPGKYI	HLA-DRB1*01:01	0.6123	Non-allergen	Non-toxin	100	Positive	Non-inducer (−0.9)	Inducer (0.31)	1	15	9.1

Collectively, these B-cell, CTL, and HTL epitopes represent strong immunogenic candidates for the design of a safe and broadly protective multi-epitope subunit vaccine against *K. pneumoniae*.

### 3.3 Population coverage and autoimmunity screening of epitopes

**Fig. 3:**
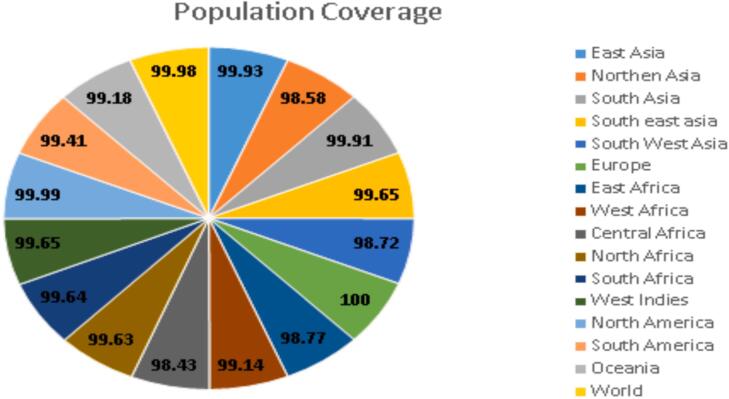
Global population coverage analysis of selected epitopes. The world map visualization demonstrates predicted vaccine coverage across geographic populations (99.98% global coverage) for major geographic regions (Africa 99.2%, Asia 99.5%, Europe 98.8%, Americas 99.1%).

To assess the global applicability of the selected epitopes, the IEDB Population Coverage Tool was utilized to analyze the distribution of both CTL and HTL epitopes derived from the IucA/IucC and FyuA proteins across various human populations. The results demonstrated exceptionally broad coverage, with a cumulative worldwide population coverage of 99.99 % (Fig. 3). This extensive representation across multiple ethnic and geographic groups highlights the universal immunogenic potential of the multi-epitope vaccine construct.

Such wide-ranging coverage ensures that the designed vaccine is likely to stimulate a robust immune response in diverse populations, supporting its application as a global prophylactic solution against *K. pneumoniae*.

To ensure safety and specificity, all selected epitopes were subjected to homology screening against the human proteome. The absence of significant similarity confirmed the non-homologous nature of the vaccine components, thereby minimizing the risk of autoimmune cross-reactivity. This step is crucial in vaccine design, ensuring that the epitopes are highly immunogenic yet non-self, reducing the potential for adverse immune responses.

Overall, the combined results of the population coverage analysis and human proteome homology screening affirm that the proposed multi-epitope vaccine is both widely effective and inherently safe for diverse global populations.

### 3.4 Vaccine design complexity and specificity

**Fig. 4:**
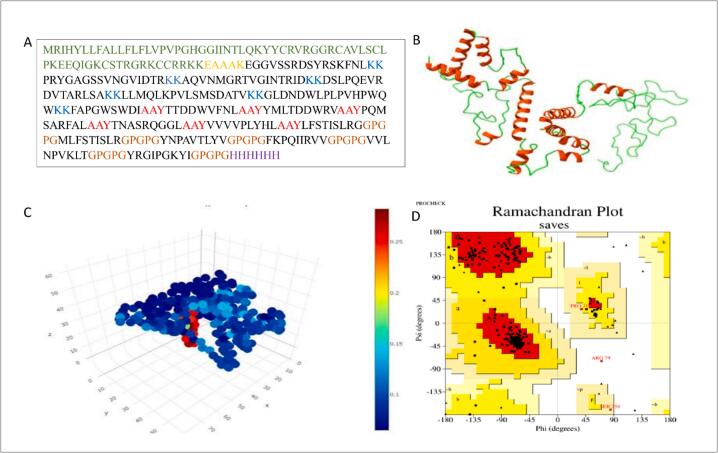
(A) Schematic representation of the vaccine construct, highlighting the β-defensin adjuvant (dark green), EAAAK linker (yellow), B-cell, CTL, and HTL epitopes (black), AAY linkers (red), GPGPG linkers (brown), KK linkers (blue), and the 6xHis tag (purple). (B) Predicted 3D structure of the NUBRI-K vaccine construct generated using the 3Dpro program. (C) PSICA server evaluation displaying a global score of 0.1093 and a MUfoldQ score of 0.9854, indicating high structural quality and stability. Blue and green regions represent areas of high confidence and structural stability, while yellow to red regions correspond to lower-confidence areas, potentially indicating flexibility, disorder, or regions requiring refinement. (D) Ramachandran plot analysis showing 91% of residues in favored regions, confirming the structural validity of the vaccine model. (For interpretation of the references to color in this figure legend, the reader is referred to the web version of this article.)

The final multi-epitope vaccine construct comprised 342 amino acids, integrating 18 immunodominant peptide sequences linked by four distinct linkers—EAAAK, KK, AAY, and GPGPG. These linkers were strategically incorporated to ensure proper spatial separation of epitopes, enhance protein folding, and improve epitope exposure and immune processing (Fig. 4A). The three-dimensional (3D) structure of the vaccine was predicted using 3Dpro, which generated three high-quality models. Model 1 was selected for further refinement based on its superior structural assessment scores (Fig. 4B). Structural quality was evaluated using PSICA, yielding a global score of 0.1093 and a MUfoldQ score of 0.9854, both indicating high model accuracy and reliability (Fig. 4C). To validate stereochemical properties, a Ramachandran plot analysis was conducted. The results showed that 91 % of residues resided in favored regions, confirming that the predicted model adopts a thermodynamically stable and immunologically relevant conformation (Fig. 4D). This high percentage of favorable residues supports the structural integrity and immunogenic potential of the vaccine construct. Collectively, the modeling and validation steps affirm that the designed multi-epitope vaccine is structurally sound, biophysically stable, and suitable for downstream immunological applications and expression studies.

### 3.5 Evaluation of vaccine candidate: antigenicity, stability, and structural features

**Fig. 5:**
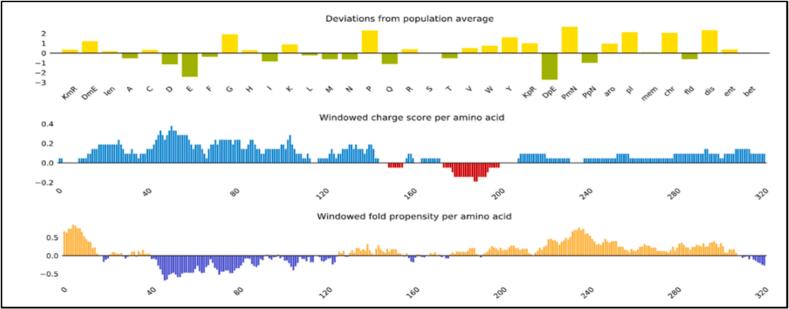
Solubility result of the vaccine construct as obtained by protein Sol server. The solubility of the vaccine construct was shown to be 0.477 compared to 0.45 of the population average solubility by *E.coli***.**

The constructed vaccine exhibited favorable physicochemical characteristics essential for a successful vaccine candidate. It had a molecular weight of 37,496.69 Da and a basic theoretical isoelectric point (pI) of 10.07, indicating a net positive charge under physiological conditions. The vaccine showed strong in vivo stability, with an estimated half-life of 30 h in human reticulocytes, over 20 h in yeast, and over 10 h in E. coli. The aliphatic index of 83.25 suggested high thermostability, while the instability index of 27.93 classified it as a stable protein. A GRAVY score of 0.205 indicated that the vaccine is overall hydrophilic, which is beneficial for solubility and interaction with the immune system. Furthermore, a solubility score of 0.477 predicted a high probability of soluble expression in recombinant systems, supporting its ease of production and purification (Fig. 5). Secondary structure prediction revealed 27 % alpha-helices, 22.22 % extended strands, 5.56 % beta-turns, and 44 % coils (Fig. S2). Conformational B-cell epitope mapping via ElliPro identified six discontinuous epitopes, with scores ranging from 0.6 to 0.746, indicating moderate to high immunogenic potential (Table 5; Fig. S3).

**Table 5:** The selected conformational B-cell epitopes using ElliPro server.

No	Residues	Number of residues	Score
1	:M1,_:R2,_:I3,_:H4,_:Y5,_:L6,_:L7,_:G38,_:R39,_:S44,_:P47,_:K48,_:E50,_:Q51,_:I52,_:G53,_:K54,_:C55,_:S56,_:T57,_:R58,_:G59,_:R60,_:K61,_:C62,_:C63,_:R65,_:K66,_:E68,_:A69,_:A70,_:A71,_:K72,_:G74,_:G75,_:V76,_:S77,_:S78,_:R79,_:D80,_:S81,_:Y82,_:R83	43	0.746
2	:K150,_:L153,_:S154,_:M155,_:S156,_:D157,_:A158,_:T159,_:V160,_:K161,_:K162,_:G163,_:L164,_:D165,_:N166,_:D167,_:W168,_:L169,_:P170,_:L171,_:P172,_:V173,_:H174,_:P175,_:Q177,_:W178,_:K179,_:W185,_:S186,_:W187	30	0.73
3	_:A224,_:L225,_:A226,_:A227,_:Y228,_:T229,_:N230,_:A231,_:S232,_:R233,_:Q234,_:G235	12	0.723
4	_:L253,_:F254,_:S255,_:I257,_:L268,_:F269,_:S270,_:T271,_:I272,_:S273,_:L274,_:R275,_:G276,_:P277,_:G278,_:P279,_:G280,_:Y281,_:N282,_:P283,_:A284,_:V285,_:T286,_:L287,_:Y288,_:V289,_:G290,_:P291,_:G292,_:P293,_:G294,_:F295,_:K296,_:P297,_:Q298,_:I299,_:I300,_:R301,_:V302,_:V303,_:G304,_:P305,_:G306,_:P307,_:G308,_:V309,_:V310,_:L311,_:N312,_:P313,_:V314,_:K315,_:L316,_:T317,_:G318,_:P319,_:G320,_:P321,_:G322,_:Y323,_:R324,_:G325,_:I326,_:P327,_:G328,_:K329,_:Y330,_:I331,_:G332,_:P333,_:G334,_:P335,_:G336,_:H337,_:H338,_:H339,_:H340	77	0.704
5	_:I189,_:A190,_:A191	3	0.683
6	_:I103,_:D104,_:T105,_:K107	4	0.601

### 3.6 Docking analysis and selection of optimal TLR4-vaccine complex model

**Fig. 6:**
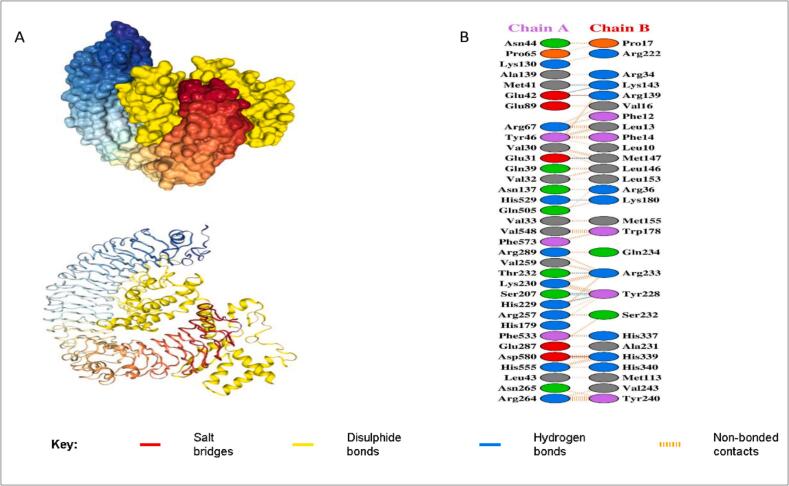
Molecular Docking Analysis of the NUBRI-K Vaccine with Toll-Like Receptor 4 (TLR4). (A) Docked complex of the NUBRI-K vaccine (yellow) with the TLR4 receptor (blue, red, and brown), illustrating the binding interaction. (B) Close-up view of the interaction interface between TLR4 (chain A) and the NUBRI-K vaccine (chain B), showing key binding residues. (C) Interaction analysis revealing eight hydrogen bonds and two salt bridges, indicating strong binding affinity (−278.22 kcal/mol) and stable complex formation. (For interpretation of the references to color in this figure legend, the reader is referred to the web version of this article.)

Protein–protein docking was conducted using ClusPro, HDOCK, and HADDOCK to assess the interaction between the designed vaccine and the TLR4 receptor. Docking results were evaluated based on binding energy, cluster size, and interaction stability. ClusPro generated multiple docking clusters (Fig. 6A), with Cluster 0 being the most populated, indicating a highly reliable binding mode. The lowest-energy structure within Cluster 0 exhibited a binding energy of –1383.2 kcal/mol, suggesting a strong and stable interaction between the vaccine and TLR4.

Similarly, HDOCK produced 10 docked complexes, with Model 1 demonstrating the lowest energy score (–278.22 kcal/mol) and the most favorable interaction profile, making it the top candidate for further analysis (Table 6). HADDOCK docking further refined the complex, yielding a docking score of –139.7 ± 3.0 kcal/mol (Table 7), consistent with strong binding affinity.

**Table 6:** Docking Results of the TLR4-Vaccine Complex Using ClusPro and HDOCK.

Software	Model	Cluster Size	Lowest Energy Score (kcal/mol)	Confidence Score	Ligand RMSD (Å)
ClusPro	0 (Best Model)	51	−1383.2	N/A	N/A
HDOCK	1 (Best Model)	N/A	−278.22	0.9285	4.5

**Table 7:** HADDOCK Docking Results for the TLR4-Vaccine Complex.

HADDOCK software	Score
HADDOCK score	−139.7 ± 3.0
Cluster size	32
RMSD from the overall lowest-energy structure	0.6 ± 0.4
Van der Waals energy	−65.1 ± 4.8
Electrostatic energy	−259.5 ± 21.2
Desolvation energy	−40.9 ± 1.5
Restraints violation energy	182.6 ± 47.5
Buried Surface Area	2355.1 ± 153.2
Z-Score	−2.2

Interaction analysis of the top-ranked TLR4–vaccine complex (Model 1) using PDBsum revealed 34 TLR4 residues interacting with 28 residues from the vaccine (Fig. 6B). Eight hydrogen bonds and two salt bridges were identified, contributing to the complex's stability (Table 8). Binding free energy calculated via the PRODIGY web server showed a ΔG of –11.4 kcal/mol and a dissociation constant (Kd) of 4.5 nM, indicative of high binding affinity and strong interaction at the nanomolar level.

**Table 8:** The result of the interaction between the chain A (TLR-4 receptor) and chain B (NUBRI-K vaccine).

Chain	No of interface residues	Interface area (Å2)	No of salt bridge	No of disulphide bonds	No of hydrogen bonds	No of non-bonded contact
A	34	1796	2	−	8	246
B	28	1984				

Collectively, the docking results from ClusPro, HDOCK, and HADDOCK consistently demonstrated a strong, stable, and biologically relevant interaction between the TLR4 receptor and the designed vaccine, supporting its potential as an effective immune-stimulating candidate.

### 3.7 Evaluation of the vaccine-receptor product by Normal Mode analysis (NMA)

**Fig. 7:**
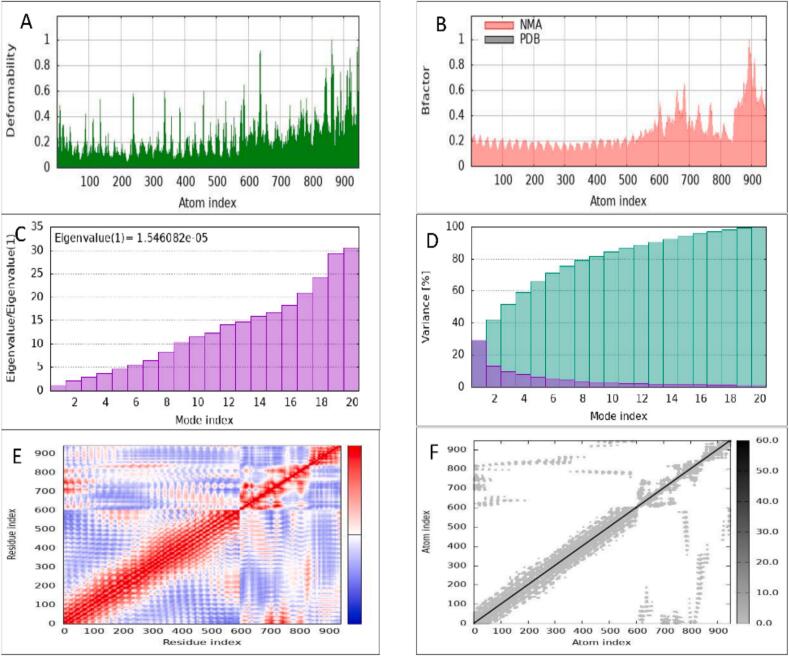
Results of normal mode analysis of vaccine SS-MEVC and TLR4 complex acquired by the iMODS server. (A) Deformability, (B) B-factor (C) eigenvalue, and (D) colored bars showing the individual (purple) and cumulative (green) variances. (E) Covariance matrix indicating correlated (red), uncorrelated (white), and anti-correlated (blue) motions of paired residues and (F) the elastic network model of the SS-MEVC-TLR4 complex (grey regions indicating stiffer regions). (For interpretation of the references to color in this figure legend, the reader is referred to the web version of this article.)

The structural flexibility and stability of the NUBRI-K–TLR4 docked complex were evaluated using Normal Mode Analysis (NMA). The deformability plot (Fig. 7 A) revealed flexible regions with peak values ranging from 0.8 to 1.0, corresponding to residues undergoing significant conformational changes that are essential for receptor–ligand interactions.

The B-factor graph (Fig. 7 B) compared NMA-derived mobility with PDB data, confirming that the complex maintains overall structural stability. An eigenvalue of 1.546082e-05 (Fig. 7 C) was obtained, indicating that the complex can undergo large, collective motions with minimal energy expenditure—an important characteristic for biologically relevant functions such as ligand binding and allosteric regulation. Variance analysis (Fig. 7 D) demonstrated that low-frequency modes, which correspond to low eigenvalues, exhibit high variances, capturing the most functionally significant movements.

The covariance map (Fig. 7 E) provided insight into the dynamic correlations between atomic movements. Red zones represent positively correlated motions, suggesting coordinated structural or functional relationships between residues. White zones denote uncorrelated regions, while blue zones indicate negatively correlated motions, where residues move in opposite directions. These patterns underscore cooperative interactions that enhance the stability and functional dynamics of the complex.

Finally, the elastic network model (Fig. 7 F) illustrated a balance between rigid and flexible regions. Darker gray areas indicated structural rigidity, offering mechanical support, whereas lighter regions represented flexibility required for conformational changes during receptor activation. Together, these dynamic and structural features support the functional integrity of the NUBRI-K–TLR4 complex and its potential role in immune activation.

### 3.8 Structural dynamics of the Vaccine–TLR4 complex (CABSflex 2.0 Analysis)

The structural flexibility of the TLR4–vaccine complex was evaluated using the CABSflex 2.0 server, which simulates residue-level dynamics through root mean square fluctuation (RMSF) profiling.

TLR4 Receptor (Chain A; Residues A28–A578):

RMSF analysis of TLR4 revealed distinct region-specific flexibility:

Stable Binding Interface (RMSF < 1.5 Å): Residues A150–A250, corresponding to the predicted vaccine-binding interface, exhibited minimal fluctuation, indicating strong and stable interactions. This stability supports previous findings from docking simulations (ΔG = −278.22 kcal/mol), underscoring a high-affinity binding interaction.

Moderately Flexible Regions (RMSF 2–3 Å): Loop regions, particularly A300–A350, showed moderate flexibility. These regions likely contribute to conformational adaptability, facilitating efficient receptor-ligand engagement during immune recognition.

**Fig. 8:**
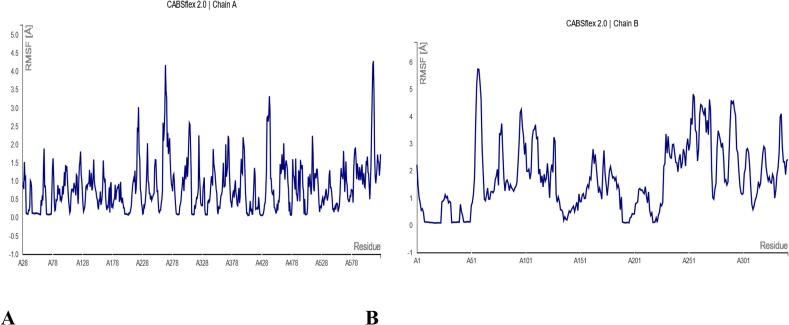
(A) Chain A (TLR4 receptor): Root mean square fluctuation (RMSF) plot showing residue flexibility (Å) across the TLR4 structure (residues A28-A578). Key binding interface regions (A150-A250) exhibit low fluctuations (<1.5 Å, blue), while terminal domains show higher flexibility (>3 Å, red). (B) Chain B (Vaccine construct): RMSF profile of the multi-epitope vaccine (residues A1-A301), with rigid adjuvant core (A1-A50, <1.5 Å) and flexible epitope regions (A51-A201, 1.5–3 Å). Gray shaded areas indicate linker sequences. Analyses performed using CABSflex 2.0 with default parameters. (For interpretation of the references to color in this figure legend, the reader is referred to the web version of this article.)

Highly Flexible Termini (RMSF >3 Å): Elevated fluctuations were observed at the N- and C-terminal ends (A28–A50 and A500–A578), consistent with their solvent exposure and limited involvement in complex stabilization (Fig. 8 A).

Multi-Epitope Vaccine Construct (Chain B; Residues A1–A301):

The vaccine candidate displayed a dynamic profile indicative of structural balance between rigidity and functional flexibility:

Rigid Adjuvant Core (RMSF <1.5 Å): The β-defensin adjuvant domain (A1–A50) exhibited structural rigidity, reinforcing its role in mediating stable interactions with TLR4 and initiating immune responses.

Moderately Flexible Epitope Regions (RMSF 1.5–3 Å): Linker regions (A51–A101) and predicted epitope clusters (A151–A201) showed moderate flexibility, which is favorable for effective antigen presentation and immune cell accessibility without compromising overall structural stability.

Highly Flexible C-terminal Segment (RMSF >3 Å): The His-tag region (A251–A301) was highly mobile, aligning with its expected unstructured and non-immunogenic characteristics (Fig. 8 B).

### 3.9 Immune simulation

**Fig. 9:**
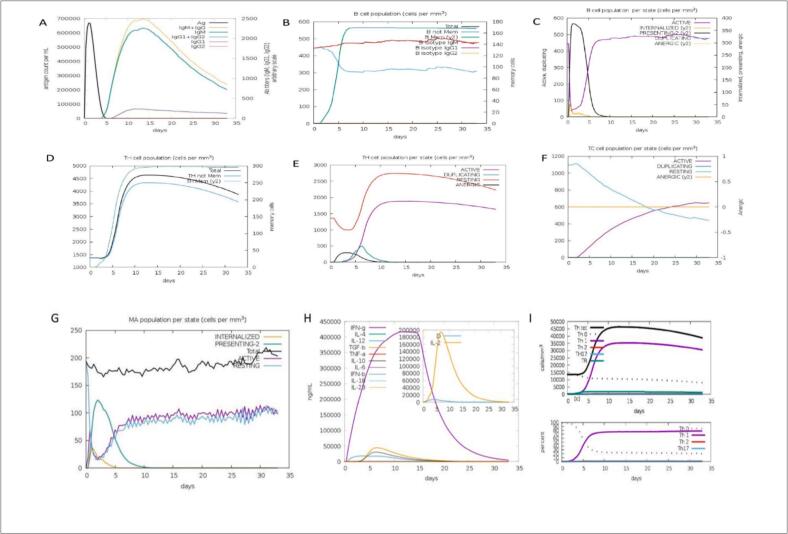
Immune simulation results of the NUBRI-K vaccine using the C-ImmSim server.. (A) IgM and IgG levels peak after the second dose, indicating strong humoral immunity.(B-C) B-cell proliferation increases with repeated immunization, supporting memory formation. (D-F) T-helper (TH) and cytotoxic T-cell (TC) populations expand significantly, suggesting robust cellular immunity. (G) Macrophage activation enhances antigen presentation. (H) IFN-γ and IL-2 production rise after the third dose, promoting a Th1-dominant response.(I) Th1 cell expansion confirms a sustained immune response, critical for long-term protection.

Immune simulation analysis (Fig. 9) demonstrated a robust and sustained immune response following vaccination with the designed construct. A marked elevation in combined IgM + IgG and IgG1 antibody levels was observed, peaking around day 15 and subsequently stabilizing above baseline levels (Fig. 9 A), indicating effective antigen recognition and clearance.

B cell populations exhibited significant post-vaccination expansion (Fig. 9 B and 9C), with enhanced proliferation and antigen presentation, suggesting a strong and well-coordinated humoral immune response. Additionally, helper T cell (TH) and cytotoxic T cell (TC) populations showed substantial increases (Fig. 9 D–F), indicating the development of robust immunological memory and long-term cellular immunity.

Innate immune activation was supported by elevated macrophage activity and increased antigen presentation (Fig. 9). Furthermore, cytokine profiling revealed a sharp rise in IFN-γ and a moderate increase in IL-2 levels (Fig. 8H), both of which are critical for T cell activation and immune regulation. Notably, a strong Th1-biased response (Fig. 9 I) was observed, indicating potent cell-mediated immunity—a key factor for effective pathogen clearance.

Collectively, these simulation results suggest that the candidate vaccine elicits a well-balanced and potent immune response, encompassing both humoral and cellular arms, and holds strong potential for providing long-term immunological protection against infectious agents.

### 3.10 Codon optimization, reverse translation, and in silico cloning of the vaccine construct

The amino acid sequence of the designed multi-epitope vaccine was reverse translated into a corresponding DNA sequence, which was subsequently optimized for expression in mammalian systems. Codon usage adaptation was performed using JCat, yielding a Codon Adaptation Index (CAI) of 1.0, indicative of perfect adaptation to the codon usage bias of human cells. This high CAI value suggests enhanced translational efficiency and protein yield in mammalian expression systems.

The GC content of the optimized nucleotide sequence was 48.27 %, which falls within the ideal range (40–60 %) for mRNA stability and optimal transcriptional activity in mammalian hosts.

**Fig. 10:**
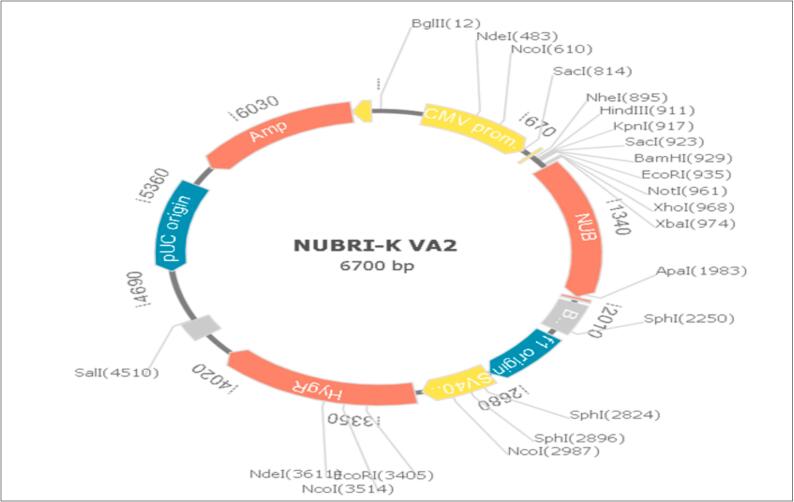
NUBRI-K vaccine in silico restriction cloning into the mammalian expression vector pcDNA3 between the XboI and ApaI restriction sites. Vaccine construct is depicted as NUB in red color.

For in silico cloning, the optimized vaccine gene was successfully inserted into the pcDNA3 mammalian expression vector, driven by the cytomegalovirus (CMV) promoter to ensure strong transcriptional activity. Cloning was performed using XbaI and ApaI restriction sites, enabling precise and directional insertion of the vaccine construct (Fig. 10). This cloning strategy supports downstream experimental validation and expression in mammalian cell lines for functional assays and immunogenicity studies.

## 4 Discussion

The rise of multidrug-resistant (MDR) *K. pneumoniae* strains poses a significant global health threat, especially in low- and middle-income countries. Chimeric vaccines, developed using immunoinformatics and vaccinomics approaches, have shown promise in protecting against various bacterial pathogens, including *K. pneumoniae*.^36^ These advanced computational techniques provide a robust framework for designing vaccine candidates with strong immunogenic and antigenic properties.

To effectively combat the ongoing threat of MDR strains, continuous monitoring of strain evolution, epitope conservation, and population coverage is essential. These strategies are critical for developing effective vaccines and managing persistent bacterial threats.^37^

Previous vaccine efforts against *K. pneumoniae* have predominantly focused on capsular polysaccharides (CPS), outer membrane proteins (OMPs), and lipopolysaccharides (LPS), which, despite their immunogenic potential, suffer from high genetic variability and frequent antigenic shifts, limiting their cross-strain efficacy.^38^ In contrast, our study introduces a rationally designed multi-epitope vaccine that targets highly conserved iron acquisition proteins FyuA and IucA/IucC identified from a comprehensive analysis of 5,727 proteins in the NUBRI-K strain as optimal vaccine targets due to their high antigenicity, essential roles in iron acquisition, and strong conservation across pathogenic strains.

Iron acquisition is vital for bacterial survival and virulence, making these proteins attractive for vaccine development. FyuA, an outer membrane receptor, is surface-exposed and readily accessible to the host immune system, enhancing its potential for antibody-mediated targeting.^39^ Furthermore, these proteins are minimally expressed by commensal bacteria, reducing the likelihood of off-target immune responses. FyuA has been previously validated as a vaccine candidate in Escherichia coli models,^40^ while IucA/IucC’s role in aerobactin biosynthesis supports its immunogenic potential.^41^ Collectively, these findings highlight FyuA and IucA/IucC as promising components of a broadly protective *K. pneumoniae* vaccine.

To assess strain coverage, we conducted a conservation analysis across 100 genetically diverse *K. pneumoniae* strains, including various sequence types (STs) and known hypervirulent lineages. FyuA and IucA/IucC demonstrated 99.9 % and 100 % conservation, respectively, indicating their potential to confer broad protection across diverse capsule types, including those associated with hypervirulence. Targeting these conserved iron acquisition systems provides a strategic advantage by circumventing the challenge of capsular variability a major obstacle in vaccine design. Future studies should incorporate opsonophagocytic assays and in vivo challenge models to further evaluate the protective efficacy of these antigens.

The selected T-cell epitopes were highly conserved and predicted to be strongly immunogenic, making them ideal for broad-spectrum vaccine development. Notably, this study identified MHC class II epitopes capable of inducing IFN-γ production, a key cytokine involved in the clearance of *K. pneumoniae*, especially hypervirulent strains.^42^ IFN-γ plays a central role in orchestrating the innate immune response during pulmonary infections.^43^ These prioritized epitopes hold strong potential for eliciting specific, effective, and long-lasting immune responses while minimizing undesirable effects.

Multi-epitope vaccines can stimulate both humoral and cellular immunity, offering advantages over traditional monovalent vaccines.^44^ Our construct incorporates validated cytotoxic T lymphocyte (CTL) and helper T lymphocyte (HTL) epitopes with high binding affinities to common HLA alleles such as HLA-A*02:01 and HLA-DRB1*01:01. The current formulation exhibits exceptional global population coverage, estimated at 99.98 %, supporting its potential as a universal vaccine candidate against MDR *K. pneumoniae*.

To enhance immunogenicity and structural integrity, we employed a rational linker design: KK linkers for B-cell epitopes, AAY for CTL epitopes, and GPGPG for HTL epitopes.^6^, ^45^ Additionally, an α-helix-forming EAAAK linker was added to both termini of the adjuvants. According to George and Heringa, natural α-helical linkers enhance structural stability and functional domain separation.^45^ Their rigidity, due to tight hydrogen bonding and compact structure, makes them well-suited for complex vaccine constructs.

The β-defensin adjuvant was incorporated due to its known ability to activate TLR4-mediated immune pathways. Docking analysis confirmed its efficacy, with a binding energy of −278.22 kcal/mol, and molecular simulations supported its role in inducing a Th1-skewed immune response. β-defensin functions both as an antimicrobial peptide and as a modulator of innate immunity, activating dendritic cells, monocytes, and T cells.^32^ A 6 × His tag (HHHHHH) was added at the C-terminal end to facilitate purification and experimental validation.^46^

Together, the inclusion of this adjuvant, selected linkers, and epitopes contributed significantly to the vaccine’s structural and immunological robustness. The final construct exhibited a favorable instability index (27.93) and solubility score (0.477). Physicochemical analysis further confirmed its stability, hydrophilicity, and basic nature.

Structural validation showed 91 % of residues within favored regions of the Ramachandran plot. The ProSA Z-score aligned with native-like structures, and a PSICA global confidence score of 0.1093 supported the model's reliability.

During *K. pneumoniae* infection, Toll-like receptors (particularly TLR2 and TLR4) are overexpressed in airway epithelial cells and play a key role in initiating the innate immune response. Accordingly, we evaluated our vaccine for TLR4 binding affinity.^1^ By combining innate immunity activation via β-defensin–TLR4 interaction and adaptive immunity via HLA-mediated epitope presentation, the vaccine is expected to elicit robust humoral and cellular responses.

Molecular docking confirmed strong and stable vaccine–TLR4 interactions. HDOCK predicted a high-affinity interaction (ΔG = −278.22 kcal/mol), stabilized by eight hydrogen bonds and two salt bridges. ClusPro predicted a robust complex (binding energy = −1383.2 kcal/mol) with a buried surface area of 2,355.1 Å^2^. HADDOCK refinement yielded a docking score of −139.7 ± 3.0 kcal/mol. Binding free energy (ΔG = −11.4 kcal/mol) and a low dissociation constant (Kd = 4.5 nM) confirmed high-affinity nanomolar binding supporting efficient immune activation.^47^

Normal Mode Analysis (NMA) of the NUBRI-K–TLR4 complex revealed low eigenvalues, indicating high structural flexibility and effective receptor binding. To address NMA’s limitations in capturing dynamic behavior, we applied CABS-flex 2.0 for coarse-grained molecular dynamics, supported by HADDOCK refinement and experimental B-factor alignment. These analyses confirmed both temporal and structural stability. The β-defensin adjuvant exhibited a stable core (RMSF <1.5 Å), essential for TLR4 activation, while epitope-rich regions showed moderate flexibility (RMSF 1.5–3 Å), which supports efficient MHC presentation. This combination of rigidity in receptor-binding regions and flexibility in antigenic regions is a hallmark of successful vaccine design. However, these approaches do not replace full time-dependent molecular dynamics (MD) simulations. Future studies employing classical MD simulations will be essential to comprehensively evaluate the dynamic stability, interaction networks, and potential conformational changes of the vaccine.

Immune simulations confirmed the vaccine's immunogenic potential, indicating high antibody titers, B and T cell activation, and enhanced macrophage responses. The cytokine profile was Th1-biased, characterized by elevated IFN-γ, IL-2, and TNF-α—crucial mediators for clearing intracellular pathogens and establishing long-term immunity.

Compared to previous vaccine candidates, our construct demonstrated more sustained antibody production than a recently proposed *Staphylococcus aureus* vaccine.^48^ However, the persistence of these memory responses and the possible requirement for booster doses can only be conclusively determined through in vivo studies and clinical trials. Future experimental validation should include longitudinal assessment of memory cell populations and protective efficacy over time to establish optimal vaccination schedules.

When integrated with molecular docking, structural validation, and immune simulations, these findings strongly support the vaccine–TLR4 complex's stability and functionality. While simulations suggest durable B and T cell memory, clinical studies measuring anti-FyuA IgG levels are needed to confirm long-term protection and inform booster schedules.

Finally, the high conservation of selected epitopes, coupled with TLR4-targeted adjuvant effects, may reduce the need for frequent booster doses. To ensure efficient expression in mammalian systems, the vaccine gene was codon-optimized, achieving a codon adaptation index (CAI) of 1.0 and a GC content of 48.27 %, indicating strong transcriptional and translational efficiency. The optimized gene was successfully cloned into the pcDNA3 vector using XbaI and ApaI restriction sites, enabling downstream expression and experimental validation.^32^

The vaccine construct’s codon optimization and in silico cloning into the pcDNA3 mammalian expression vector support intramuscular (IM) administration, which is well-suited for achieving efficient in vivo antigen expression and uptake by antigen-presenting cells (APCs). This delivery route complements the β-defensin adjuvant’s activation of TLR4, collectively promoting robust systemic and cellular immune responses.^49^ However, given *K. pneumoniae*’s potential for respiratory tract colonization, future experimental studies should compare IM and intranasal routes to evaluate mucosal immunity and site-specific protection.

## 5 Study limitations

Despite the successful application of reverse vaccinology and immunoinformatics approaches, several limitations must be acknowledged to provide a balanced perspective.

First, this study relies entirely on in silico predictions-including antigenicity, immunogenicity, allergenicity, structural modeling, molecular docking, and immune simulations-without experimental validation. Consequently, the vaccine’s true immunogenic potential, safety profile, and protective efficacy remain to be confirmed through in vitro assays and in vivo animal models.

Second, although we prioritized conserved antigenic epitopes across diverse *K. pneumoniae* strains, genetic variability beyond the analyzed isolates may impact vaccine effectiveness. Additionally, computational predictions inherently carry biases and simplifications that cannot fully replicate the complexity of biological systems, such as post-translational modifications, antigen processing, vaccine delivery challenges, and human-specific immune responses.

Third, while allergenicity screening was performed to minimize adverse reactions, the potential for unexpected cross-reactivity with host proteins or commensal microbiota cannot be entirely excluded. Comprehensive experimental evaluation, including immunotoxicity and cross-reactivity assays, is essential before clinical translation.

Finally, practical considerations such as vaccine formulation, stability, scalability of production, and population-specific immune variability were beyond the scope of this study but represent critical areas for future research.

## 6 Conclusion

Currently, no vaccine exists for the prevention or treatment of *K. pneumoniae* in Sudan or globally. Given the bacterium’s ability to evade host immune responses and its genetic diversity, there is an urgent need to develop a vaccine that provides effective immunity. Genomics and bioinformatics offer powerful tools to identify suitable vaccine candidates, allowing for a rational and efficient vaccine design. This study highlights the potential of FyuA and IucA/IucC proteins, which play crucial roles in iron utilization, as promising targets for vaccine development. The conservation of selected epitopes across multiple strains suggests that the proposed vaccine could provide broad protection by triggering strong B-cell and T-cell responses, including IgM/IgG antibody production, macrophage activation, and cytokine release, particularly IFN-γ. Computational modeling, structural analysis, and simulations have confirmed that the designed vaccine is stable and capable of inducing an immune response. Additionally, codon optimization has ensured a DNA construct suitable for mammalian expression, enhancing its translational potential. However, while in silico analyses provide valuable insights, experimental validation is essential before clinical applications can be considered. Future studies must focus on in vitro and in vivo experiments to confirm immunogenicity, safety, and efficacy in animal models. Beyond immunogenic validation, manufacturing challenges must also be addressed. Large-scale production may require optimization of protein expression systems, stability in formulation, and cost-effective purification methods to ensure feasibility and accessibility. If successfully validated, the NUBRI-K vaccine could represent a significant breakthrough in combating multidrug-resistant *K. pneumoniae* infections worldwide. Despite the promising in silico findings presented in this study, several limitations must be acknowledged. First, the immunogenic potential and safety of the proposed vaccine construct remain to be validated through in vitro assays and in vivo animal models. Second, while computational methods provide rapid and cost-effective predictions, they may not fully replicate the complex biological environment. Third, although the vaccine targets highly conserved iron acquisition proteins, ongoing surveillance of *K. pneumoniae* strains is necessary to ensure continued antigenic relevance. Lastly, large-scale vaccine production may face challenges such as expression efficiency, purification, and stability under varied formulation conditions. Addressing these limitations in future studies will be crucial for progressing this vaccine candidate toward clinical application

## Declaration of Generative AI and AI-assisted technologies in the writing process

During the preparation of this work, the authors used ChatGPT to identify errors as well as to refine the clarity and coherence of the manuscript. After using this tool, the authors carefully reviewed and edited the content as needed and take full responsibility for the final version of the manuscript.

## CRediT authorship contribution statement

**Mayada M. Elfadil:** Writing – original draft, Visualization, Validation, Methodology, Formal analysis, Data curation, Conceptualization. **Samah Omer A. Samhoon:** Formal analysis. **Moaaz M. Saadaldin:** Writing – review & editing, Validation, Methodology. **Sabah A.E. Ibrahim:** Software, Resources. **Ahmed Abdelghyoum M. Mohamed:** Formal analysis. **Omnia H. Suliman:** Formal analysis. **Osama Mohamed:** Formal analysis. **Nadzirah Damiri:** Writing – review & editing. **Mohd Firdaus-Raih:** Writing – review & editing. **Sofia B. Mohamed:** Writing – review & editing, Writing – original draft, Visualization, Validation, Supervision, Methodology, Formal analysis, Conceptualization. **Qurashi. M. Ali:** Writing – review & editing.

## Funding

This project was funded by National University Biomedical Research Institute, National University-Sudan grant NUSU-2023456.

## Declaration of competing interest

The authors declare that they have no known competing financial interests or personal relationships that could have appeared to influence the work reported in this paper.

## Data Availability

The data supporting the findings of this study are available from NCBI GenBank under the assembly accession number GCA_004790705.1, which includes the 5,607 proteins of *K. pneumoniae* strain NUBRI-K (ST14). Additional datasets generated and analyzed during this study are either: Publicly accessible through relevant databases, with links provided in the manuscript. Available upon reasonable request from the corresponding author.
