# Supplementary material for: Reverse vaccinology and immunoinformatics approaches for multi-epitope vaccine design against Klebsiella pneumoniae reveal a novel vaccine target protein
Source: J Genet Eng Biotechnol. 2025 May 23;23(3):100510. doi: 10.1016/j.jgeb.2025.100510 (PMC12152629; doi:10.1016/j.jgeb.2025.100510)
Supplement: Supplementary Data 1 [file mmc1.docx]

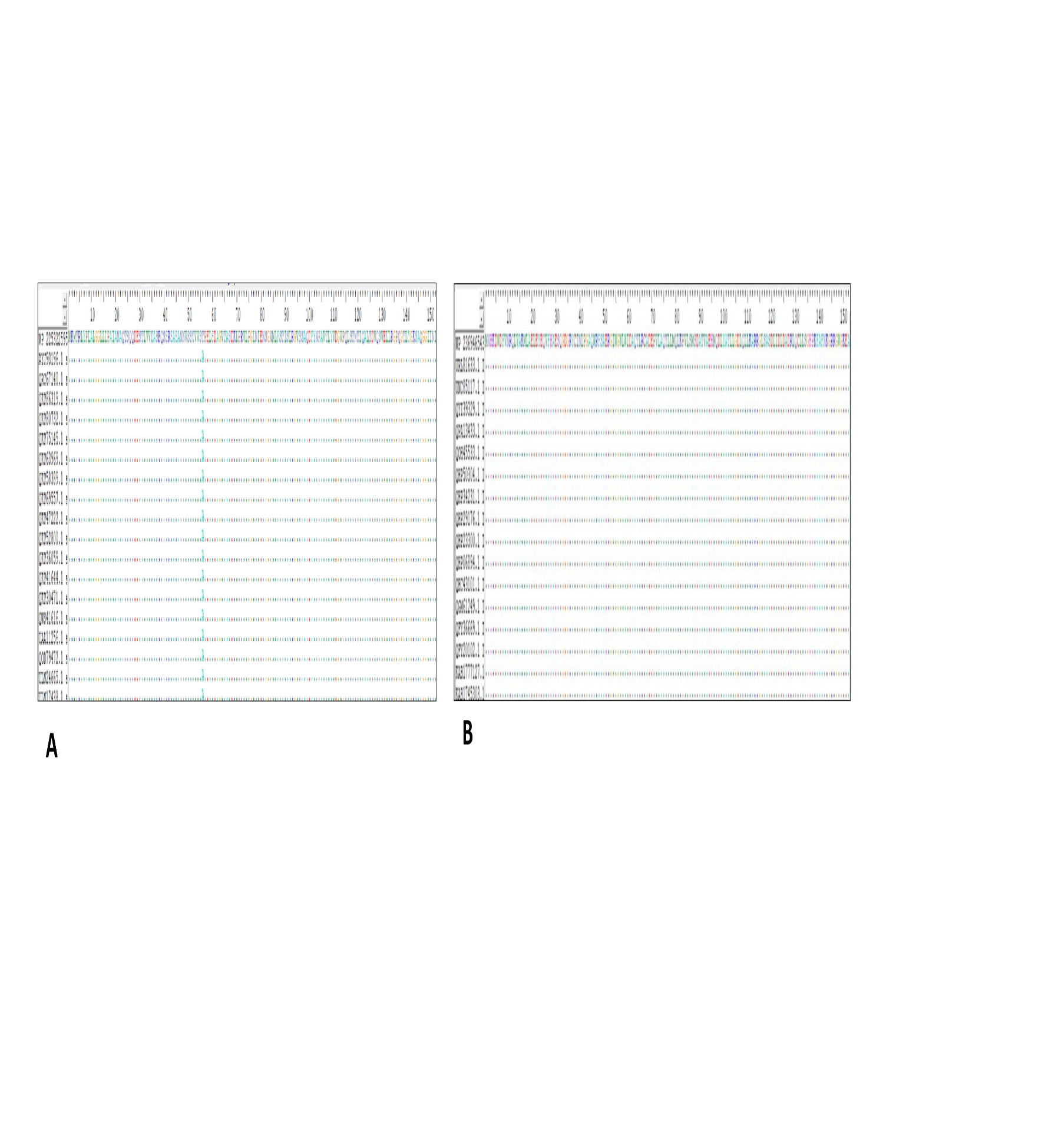


**Fig S 1:** Sequence alignment of the siderophore yersiniabactin receptor FyuA and the siderophore biosynthesis of the IucA/IucC family using ClustalW in Bioedit software. Conserved sections are represented by dots, while non-conserved portions are indicated not by letters.


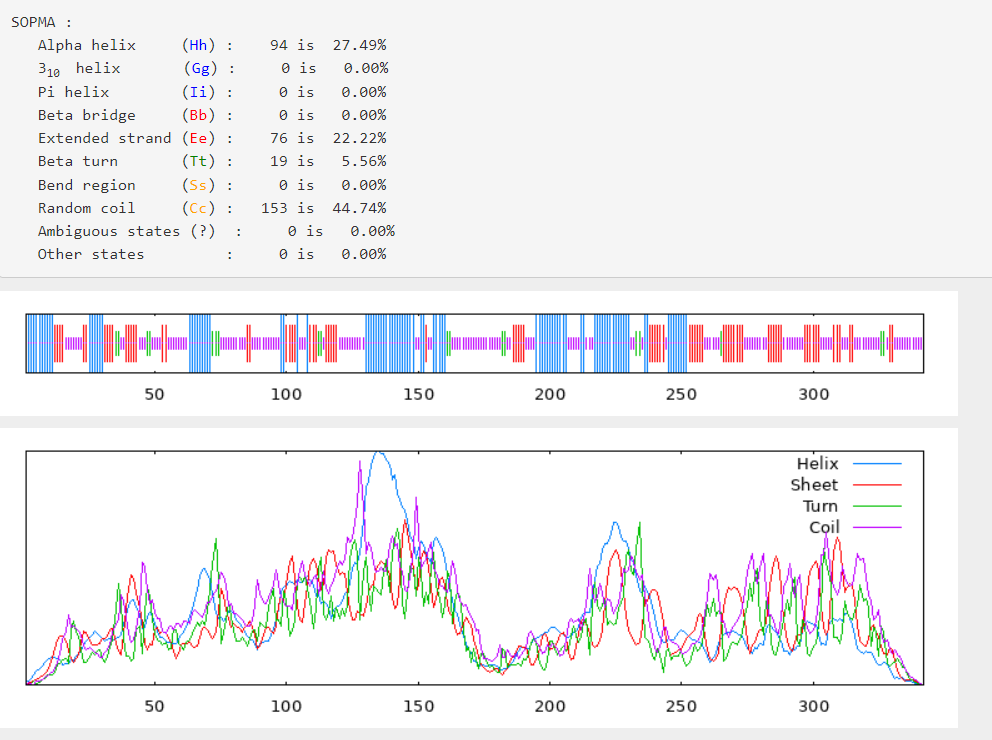


**Fig S 2:** Secondary structure prediction of vaccine construct. Alpha helices were shown in blue color, while extended strand and beta turns shown by red and green colours, respectively

**
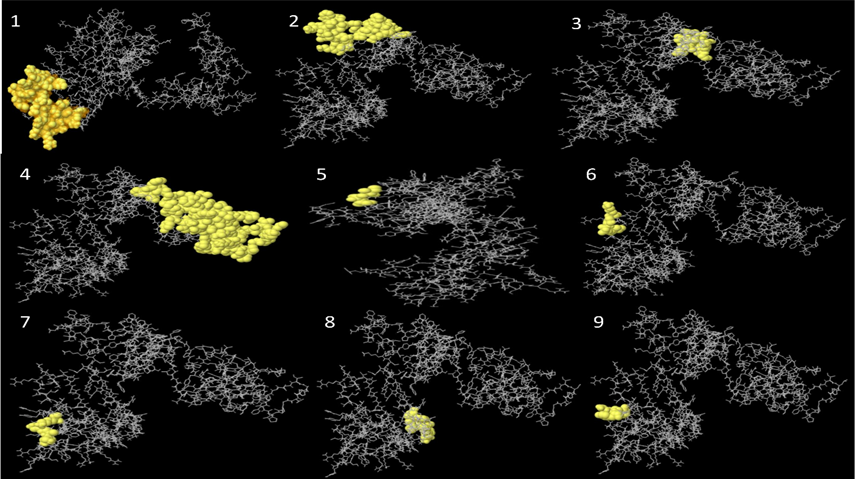
**

**Fig S 3:** Conformational B cell epitopes predicted in the NUBRI-K 3D structure. Each field corresponds to one discontinuous B cell epitope (1-9), in agreement with the results presented in Table 4.
