# Supplementary material for: Reverse vaccinology and immunoinformatics approaches for multi-epitope vaccine design against Klebsiella pneumoniae reveal a novel vaccine target protein
Source: J Genet Eng Biotechnol. 2025 May 23;23(3):100510. doi: 10.1016/j.jgeb.2025.100510 (PMC12152629; doi:10.1016/j.jgeb.2025.100510)
Supplement: Supplementary Data 2 [file mmc2.docx]

| **Accession No** | **Protein** | **TMH≤1** | **MW <110 kDa** | **Antigenicity** | **conservation** |
| --- | --- | --- | --- | --- | --- |
| TGP09655.1 | (2,3-dihydroxybenzoyl)adenylate synthase | 0 | 58.9 | 0.4024  ANTIGEN | Conserved |
| TGP09645.1 | TonB-dependent siderophore receptor | 0 | 82.3 | 0.7778  ANTIGEN | Conserved |
| TGP09653.1 | Fe2+-enterobactin ABC transporter substrate-binding protein | 0 | 34.1 | 0.4227  ANTIGEN | Conserved |
| TGP01640.1 | type 1 fimbrial protein subunit FimA | 0 | 18.1 | 0.7565  ANTIGEN | Conserved |
| TGP01639.1 | tyrosine recombinase | 0 | 23.5 | 0.5279  ANTIGEN | Conserved |
| TGP03888.1 | siderophore yersiniabactin receptor FyuA | 0 | 73.6 | 0.6656  ANTIGEN | Conserved |
| TGP00627.1 | IucA/IucC family siderophore biosynthesis protein | 0 | 64.9 | 0.4209  ANTIGEN | Conserved |
| TGP00625.1 | IucA/IucC family siderophore biosynthesis protein | 0 | 65.8 | 0.4188  ANTIGEN | Conserved |
| TGP00624.1 | lysine 6-monooxygenase | 0 | 48.1 | 0.5067  ANTIGEN | Conserved |
| TGP03878.1 | yersiniabactin biosynthesis salicylate synthase YbtS | 0 | 47.9 | 0.4256  ANTIGEN | Conserved |
|  |  |  |  |  |  |

**Table supplementary 1: presents the results of TMh, MW, antigenicity, and conservation of the ten virulence proteins.**
